# Analyzing constitutional courts as centralizers and the impact of supermajority rules: A dataset on the federalism conflicts on the unconstitutionality of Laws in Mexico

**DOI:** 10.1016/j.dib.2024.111123

**Published:** 2024-11-14

**Authors:** Mauro Arturo Rivera León

**Affiliations:** University of Silesia in Katowice, Bankowa 12, 40-007 Katowice, Poland

**Keywords:** Federalism, Supermajority rules, Municipalities, States, Separation of powers

## Abstract

The paper describes a dataset obtained through the detailed analysis of 688 judgments issued by the Mexican Supreme Court in constitutional controversies related to separation of power disputes within federalism conflicts centering on those involving the constitutionality of legislation. The data was collected in June 2022, after which judgments were extracted from the database of the Mexican Supreme Court and manually classified. With over 9000 data points, the dataset provides information such as the judgment id, the year resolved, the plaintiff, the level of government sued, the presence of the Federal District as a party, the remedy that procedurally could be sought, and the type of normative provision challenged. Furthermore, the dataset provides a time-consuming manual classification of the outcome of all challenged provisions, sorting them as upheld, invalidated, dismissed due to the supermajority requirement to strike down legislation, or dismissed on formal procedural grounds. The dataset could be of potential use to test hypotheses related to the centralizing nature of constitutional courts and other bodies resolving federalism disputes, testing the impact of supermajority rules on courts, and employing data for cross-comparison of unconstitutionality rates. The dataset has also laid a solid foundation for further annotation efforts, which may be undertaken by expanding the coded variables.

Specifications Table

**Table:** 

Subject	Law.
Specific subject area	Data from Judgments of the Mexican Supreme Court in constitutional controversies involving federalism disputes on the constitutionality of legislation.
Type of data	Table, Raw, Analyzed
Data collection	1013 judgments on constitutional controversies were analyzed from the Mexican Supreme Court database for the period 01.01.1995-21.06.2022. Subsequently, judgments not pertaining to the constitutionality of normative provisions or unrelated to federalism conflicts between different levels of government were discarded. The remaining 688 judgments were downloaded and manually classified according to thirteen variables. Coding was validated through a double-checking process, ensuring the accuracy of the classification.
Data source location	Data was collected from the Database of the Mexican Supreme Court available online: https://bj.scjn.gob.mx/
Data accessibility	Repository name: Zenodo Data identification number: DOI: 10.5281/zenodo.13992306 Direct URL to data:https://zenodo.org/records/13808059
Related research article	

## 1 Value of the Data

• The data yield insights into the resolution of federalism conflicts between the Federal Government, States, and Municipalities in Mexico, thus expanding our knowledge of conflict resolution in federalism disputes, unconstitutionality rates, the centralizing nature of constitutional review, and the impact of supermajority rules on constitutional courts resolving constitutional controversies. The dataset offers extensive information that allows for cross-jurisdiction comparison with other currently available data, providing not only national insights on one jurisdiction but also comparative possibilities.
• The dataset employs an innovative methodology that provides a detailed classification of the outcome of single normative provisions, offering nuanced insights into the outcome of cases not usually present in other datasets that classify resolutions as “invalidation” when a single provision is struck down. Such a methodology provides a new approach to study patterns of judicial decision-making and could be used to code judicial decisions in other jurisdictions.
• The dataset has laid a solid foundation to explore decision-making, federalism conflicts, and the role of supermajority rules in constitutional adjudication. Moreover, with minor subsequent annotation efforts to include additional variables, other researchers could employ the dataset to test several hypotheses of legal theory or political science, such as attitudinal model-related hypotheses.

## 2 Background

Research on federalism disputes is abundant. Recently, a segment of the literature has focused on determining whether constitutional courts and other adjudicative bodies resolving federalism disputes tend to centralize powers while resolving cases [[1], [2], [3], [4], [5], [6]]. Others have analyzed the rise of Federalism disputes in several jurisdictions [7,8]. While some research has focused on Mexico, studies have employed limited samples concerning decisions against the Federal Executive [9], focused primarily on qualitative research [9] or considered mainly cases filed without providing data for judicial outcomes [10]. Thus, there is a global trend in analyzing the centralizing function of constitutional courts and simultaneously a need for reliable data on the Mexican jurisdiction.

In addition, the Mexican Supreme Court functions with a supermajority requirement to strike down legislation [11], employing a complex system that produces *erga omnes* effects if a higher level of government brings the challenge but generates *inter partes* effects in other cases. While theoretical inquiries into the value of supermajority rules in constitutional adjudication are growing [[12], [13], [14], [15]], studies that portray their empirical impact on constitutional courts are relatively scarce, with a few exceptions [[16], [17], [18], [19]]. The dataset was also produced to allow for testing the impact of supermajority rules in constitutional adjudication of federalism disputes.

## 3 Data Description

Data was obtained using the methods and employing the coding described in the section below. The Zenodo record contains the dataset in .xlsx and .cvs files. Additionally, a ZIP file is accessible, which contains a directory with 688 MS Word files corresponding to the classified judgments. Each file has been labeled employing the judgment ID number, allowing easy access and identification of all judgments and granting the possibility of directly analyzing them qualitatively or through text analysis tools.

Data was extracted manually and classified containing the following information: 1) judgment ID number, 2) year filed; 3) year resolved; 4) number of provisions dismissed by the supermajority requirement, 5) number of provisions upheld; 6) number of provisions dismissed on formal grounds, 7) number of provisions declared unconstitutional; 8) total number of provisions challenged; 9) plaintiff; 10) level of government sued; 11) presence of the Federal District as party; 12) type of effects/remedy pursued; 13) type of normative provisions challenged.

**Fig. 1:**
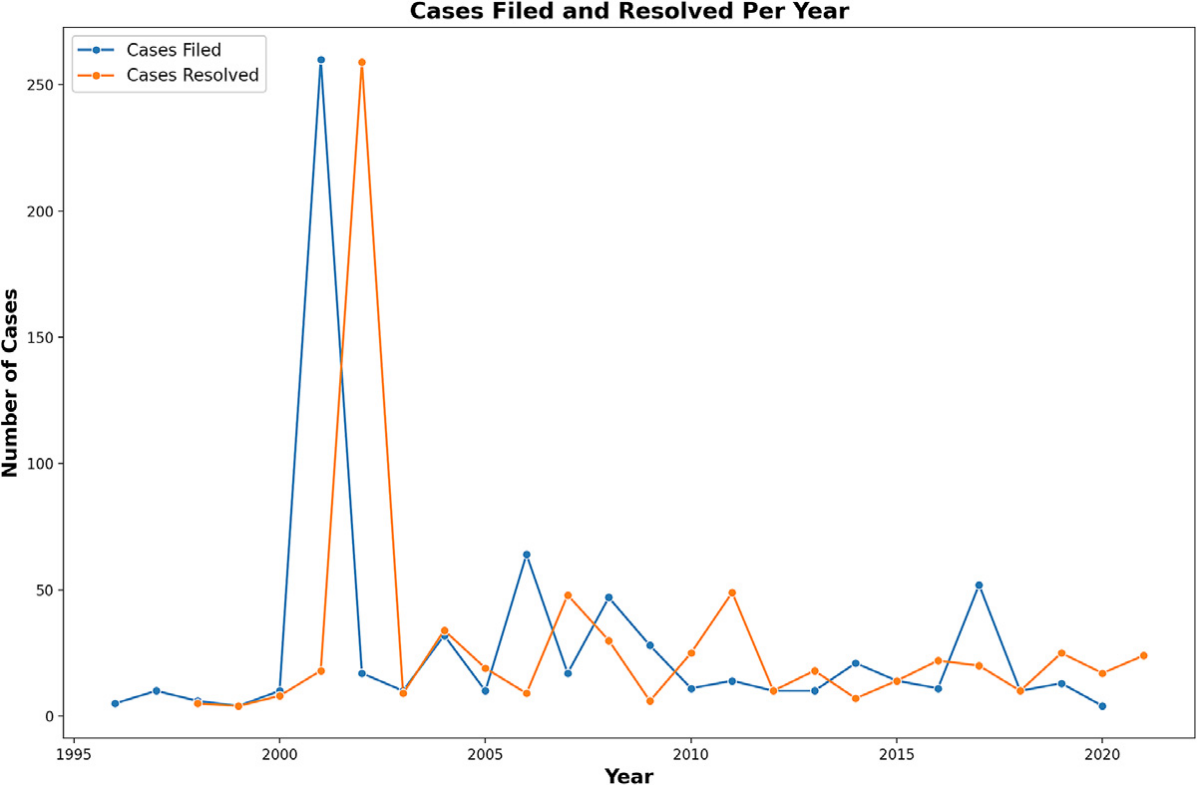
Annual trend of constitutional controversies filed and resolved by the Mexican Supreme Court (1995–2022).

Fig. 1 portrays the number of cases filed per year alongside the number of cases resolved per year. As visible from the figure, 2001 and 2002 are apparent anomalies for cases filed and resolved, respectively. The anomaly is attributable to the attempt of hundreds of municipalities to individually file constitutional controversies challenging a constitutional amendment regulating the rights of Indigenous people. All said controversies were declared inadmissible in 2002.

The dataset allows for testing multiple aspects of constitutional controversies. Having outlined in previous sections that the information can be particularly useful in testing the centralization hypothesis of constitutional courts as arbiters of federalism conflicts or the impact of supermajority rules, I will provide an overview of both through descriptive statistics.

As to the centralizing/non-centralizing nature of constitutional review in federalism disputes, given the fact that the dataset provides a specific number of provisions upheld, declared unconstitutional, procedurally dismissed, or protected by the supermajority rule, it is possible to derive unconstitutionality rates from the dataset, both generally and specifically regarding plaintiffs and defendants.

Depending on the claims brought by the parties, in some judgments, the Court was asked to analyze the constitutionality of a single or several provisions. To consider a provision as challenged, an applicant had to claim that a given “article” or “section” contravened the Constitution or an international treaty. Some judgments had as few as one single provision challenged (for example, CC 82/2017), and others had as many as 84 (CC 16/2017). Summing up all the constitutional challenges (e.g., a claim that Article 55 of the Federal Criminal Code infringes Article 19 of the Constitution), within the analyzed 688 judgments, the Court was asked to resolve the constitutionality of 6014 different normative provisions. That gives an average of 8.74 provisions per judgment. Since 742.64 provisions were declared unconstitutional, the general unconstitutionality rate is 12.34 %.

The “Experimental Design, Materials, and Methods section” offers a detailed explanation of the methodology the dataset employed to allot values (.25, .33, .50, and 1) to the different legal outcomes the Court may reach per normative provision. In a nutshell, the Supreme Court assigns individual outcomes per normative provision, i.e., a provision will be considered either constitutional, unconstitutional, dismissed, or will fail to attain the required majority to be considered unconstitutional. Each provision is voted on separately. Since the Court may declare a provision unconstitutional or constitutional, declare the challenge inadmissible for procedural reasons, or dismiss the claim due to the lack of a sufficient majority (four potential outcomes), the dataset assigned “1” to a provision that attained a single result, but assigned fractions equal to the number of achieved results when a normative provision attained two or more partial outcomes—for example, if one paragraph of a provision was considered constitutional but another paragraph was considered unconstitutional. See the relevant section for further details.

As reflected by the data, the plaintiff and the level of government sued are decisive factors in said unconstitutionality rate. There is a clear trend showing that when a higher level of government sues an inferior one, unconstitutionality rates grow. This is reflected in cases involving the following configurations: Federal Government v. State, Federal Government v. Municipality, and State v. Municipality.

**Fig. 2:**
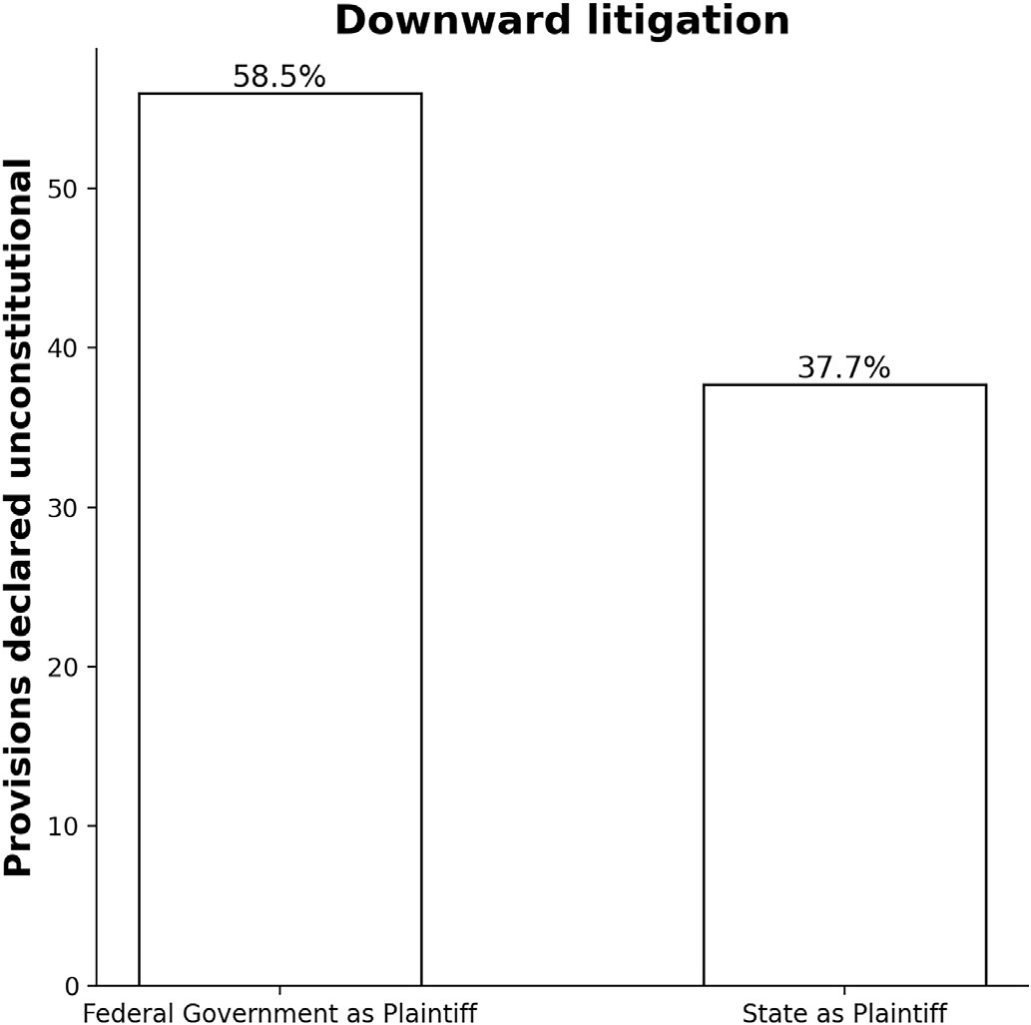
Percentage of provisions declared unconstitutional in downward litigation cases initiated by Federal and State governments.

Fig. 2 provides an overview of downward litigation, portraying higher unconstitutionality rates than the average. When the Federal Government litigates in its exclusively downward configuration, it achieves an astonishing 58.5 % unconstitutionality rate, while States attain a relatively high 37.7 %.

The unconstitutionality rates show a significant descent when considering the reverse situation. Upward litigation may occur when a lower level brings a challenge against legislation issued by a higher level, such as Municipality v. State, Municipality v. Federal Government, or State v. Federal Government.

**Fig. 3:**
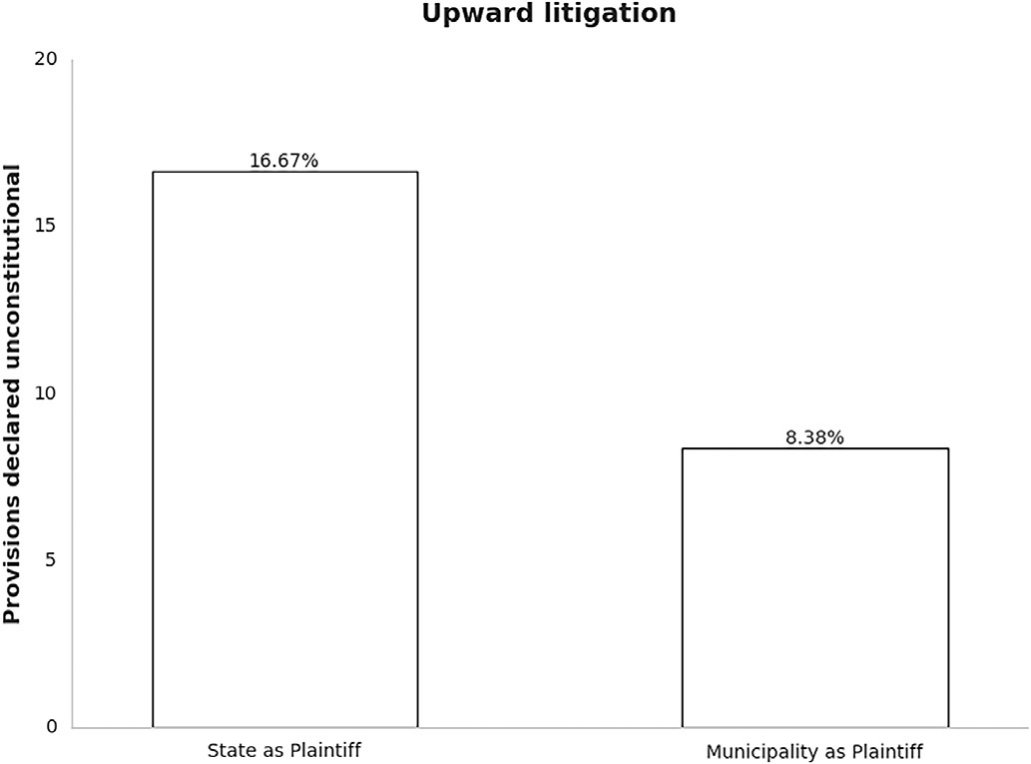
Percentage of provisions declared unconstitutional in upward litigation cases with states and municipalities as plaintiffs.

Fig. 3 exemplifies said relationship. States obtain a very modest 16.67 % unconstitutionality rate, while municipalities have one as low as 8.38 %. The data allow testing detailed scenarios of upward and downward litigation per level of government.

Of course, several factors affect the representativeness of such unconstitutionality rates even when accurately calculated. As the methods section explains, there is a substantial difference in the remedy that can be sought in constitutional controversies in upward and downward litigation, with the first pursuing only *inter partes* effects and the latter being able to generate *erga omnes* effects. *Inter partes* effects are those only affecting the parties within the judgment—for example, a law can be declared unconstitutional solely to the state or municipality that challenged it but remain valid and applicable to the remaining states and municipalities. In turn, *erga omnes* effects are those that render a declaration of unconstitutionality generally and, hence, are valid throughout the national territory, even in those states and municipalities that did not seek the remedy [20]. This procedural asymmetry creates incentives mainly for municipalities—and, to a lesser degree, states— to present identical lawsuits to be able to benefit from the same remedies.

However, given the Mexican Supreme Courtʼs practice of issuing identical judgments on identical lawsuits, researchers may account for the noise generated by duplicates. For example, statistically, it would be possible to strive to clear those anomalies by generating individual datasets without duplicates and rerunning calculations. In some cases, duplicates are easy to identify by noting a replication of the number of challenged normative provisions and of individual outcomes, usually on successive judgments within the same year. Nonetheless, in other cases, duplicates require extensive qualitative analysis to determine connected precedents or would only be detectable through textual analysis (e.g., plagiarism software). Several complications may exist. For example, the plaintiffs may have introduced one or two new challenges, or the precedent may be from a different year, or challenges may simply have not been filed consecutively in the same period. To provide an example of the influence of duplicates, I have recalculated the unconstitutionality rates of municipalities without duplicates from a well-known case, “*Reforma Indígena*.” After clearing 248 duplicates out of 249 identical decisions, cases with municipalities as plaintiffs (N=397) offer nearly 459 provisions declared unconstitutional out of 4220 challenged provisions, resulting in a 10,86% unconstitutionality rate, which is still low but slightly superior to the one previously calculated above.

Finally, it may also be interesting for scholars to analyze the impact of supermajority rules on courts to see the effects of such rules in federalism disputes. The dataset provides a meager 16 instances in which the Court failed to muster the eight-vote supermajority. The total number of provisions saved by the supermajority requirement amounts to merely 15 provisions, given the fact that some decisions pertain to partial dismissals. Thus, a provision remained valid despite the majority of the Court considering it unconstitutional in only a handful of instances. In the 6014 provisions analyzed, less than 0.3 % (0.249) were protected by the supermajority rule.

Basic descriptive analyses of unconstitutionality rates and the impact of supermajority rules based on an earlier version of this dataset were published in Spanish [21].

## 4 Experimental Design, Materials and Methods

Judgments were acquired from the Database of the Mexican Supreme Court, corresponding to constitutional controversies resolved within the period 01.01.1995-21.06.2022. The starting period matches the beginning of the functioning of the Supreme Court after the 1994 constitutional amendment, enhancing the competence of the Court to function as an arbiter of federalism conflicts. The latter period was the closing point of the previously referenced paper.

All available judgments pertaining to constitutional controversies from the period were analyzed through the Court's database viewer, having a total of 1013 judgments. To obtain the relevant sample solely pertaining to cases related to the constitutionality of normative provisions, all non-federalism conflicts were discarded (i.e., conflicts between two co-equal branches of a State or the Federal Government). Subsequently, the identification data provided by the introductory part of the judgment was employed to determine whether the plaintiff challenged the constitutionality of a normative provision.

**Fig. 4:**
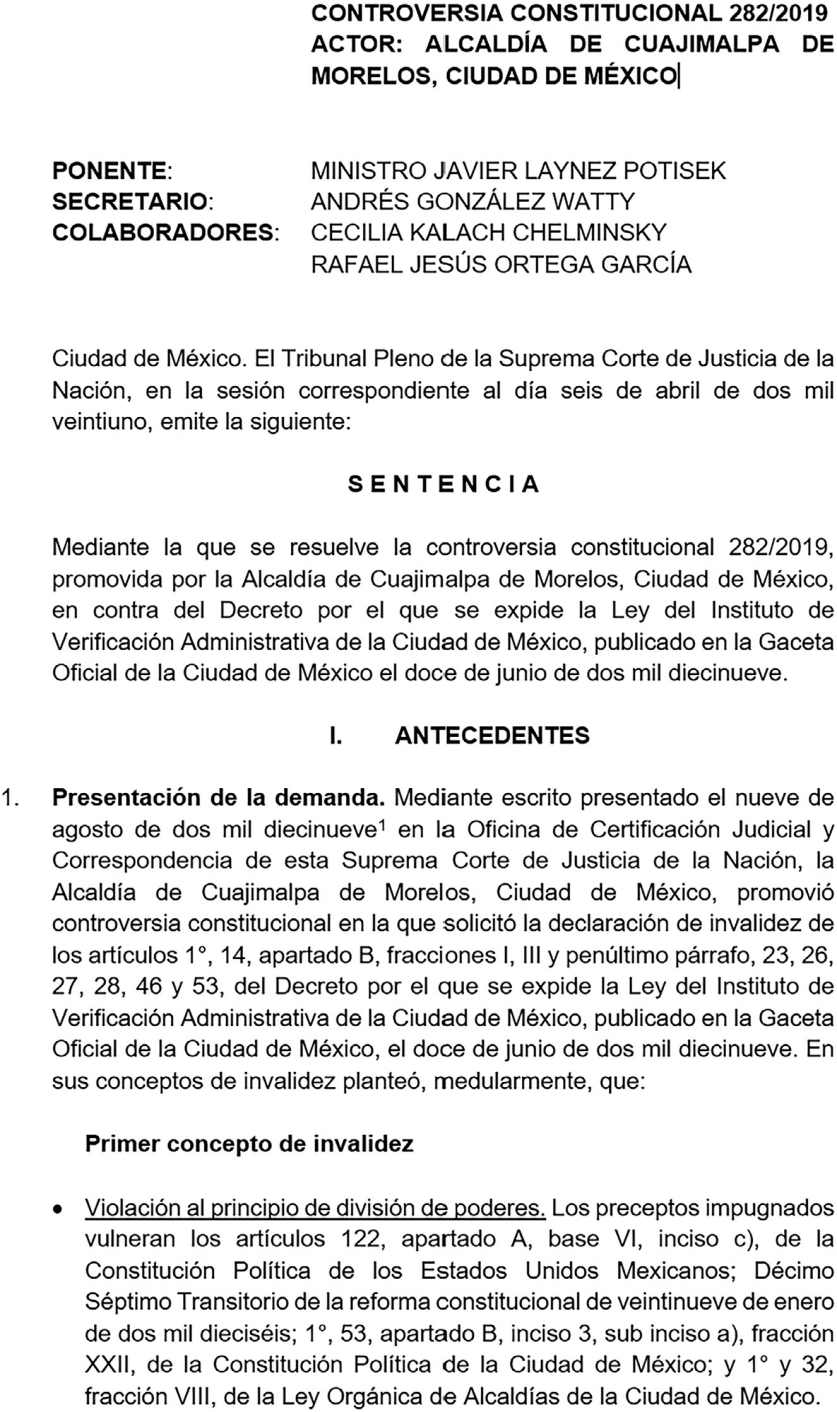
Sample identification data section in constitutional controversies (Variation 1).

**Fig. 5:**
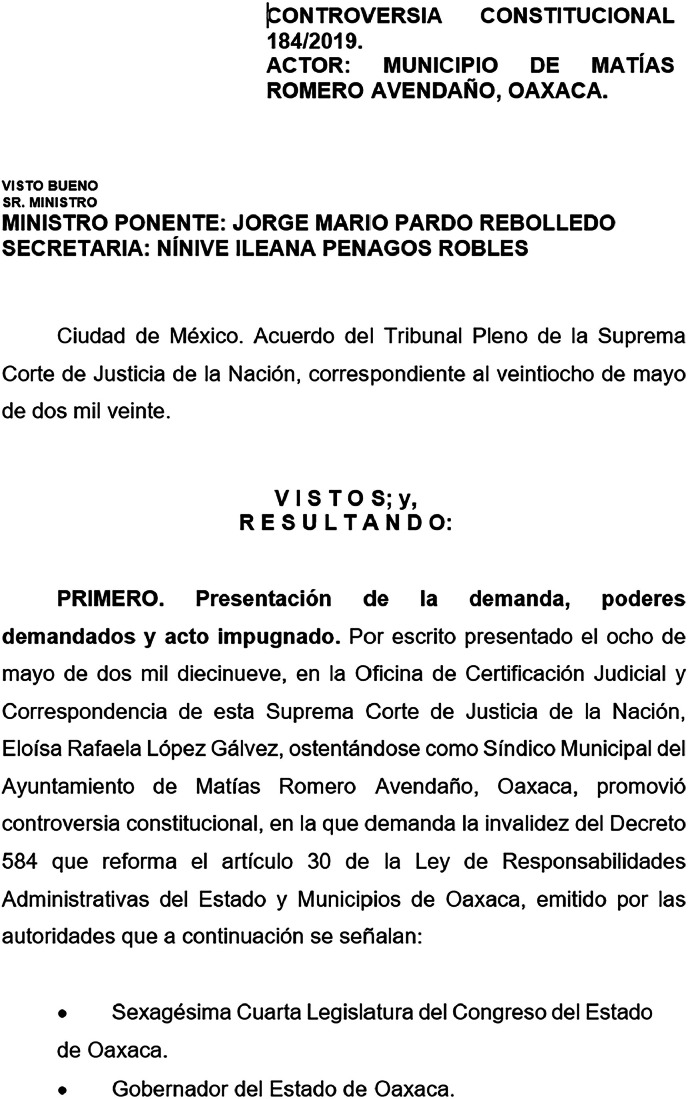
Sample identification data section in constitutional controversies (Variation 2).

Fig. 4, Fig. 5 portray two common variations on the format of Supreme Court judgments which identify in a different manner the plaintiff and the challenged provision in an introductory section of the judgment. Although sections may vary in design and denomination, the examples of CC 282/2019 and CC 184/2019 show that they are relatively straightforward to classify.

Doubts were clarified by comparing further sections of the judgment, such as “*precisión del acto impugnado*” (clarification of the challenged act) or the arguments of the plaintiff (*conceptos de invalidez*).

Manual coding was performed through the categories explained below. While referring to a different dataset, the coding of this dataset was done according to the instructions provided in the supplementary material of an earlier study [18] which classified a different set of judgments. Results were captured in a .xlsx file, which was subsequently converted to .cvs to facilitate statistical analysis.

After the relevant subsample was selected, each document was classified employing the following categories, described below at length.

1. Category 1 is the judgment ID Number. Judgments before the Mexican Supreme Court are assigned the name of the relevant procedure (i.e., *acción de inconstitucionalidad* or *amparo en revisión*), the numerical sequence of filing, and the year of filing separated by a slash. The dataset employed an abbreviated form consisting of numerical sequence-double n dash-year of filing, since all classified judgments are constitutional controversies (*controversia constitucional*). Hence, the last entry (195—2020) would be referenced as “controversia constitucional 195/2020.”

2-3. Categories 2 and 3 identify the years filed and resolved. Year filed indicates the year in which a case was formally submitted to the Court. As to the year resolved, the dataset took the year pertaining to the public session where the case was voted on. Neither the court's database nor the judgments provide information about when the “*engrose*”—the final version of the resolution containing all changes approved in the public deliberation —was made available in the court's database. This of course produces some limitations regarding the dataset, since judgements resolved previously to the closing date of the collection but published subsequently are not taken into account by the sample.

4. Category 4 indicates the number of times per judgment in which the Supreme Court was unable to invalidate a normative provision due to the lack of a sufficient majority. The Mexican Supreme Court requires a supermajority of eight votes out of eleven justices to strike down legislation. Failure to reach the prescribed threshold results in the court dismissing the challenge without formally addressing the validity of the provision, effectively deciding not to decide [18]. Thus, the variable indicates when such an outcome has occurred, namely, the fact that the supermajority rule prevented striking down a normative provision.

5-7. Category 5 provides the number of provisions upheld in a given judgment, that is, found compatible with the constitution or international treaties. Category 6 provides the number of provisions that were formally dismissed and not analyzed by the court. That is, provisions not examined by the judgment for procedural reasons that preclude the court from analyzing the case, such as lack of legal standing, mootness, failure to comply with time limits to sue, etc. Those are instances in which no formal substantive outcome was reached. Finally, category 7 indicates the number of provisions invalidated, that is, determined unconstitutional by the judgment having achieved the required eight-vote supermajority.

8 and coding values. Category 8 provides an overview of the total of provisions challenged in a given judgment. As to the coded values, most often, plaintiffs challenge an entire normative provision, which in Mexico receives the name of Article (*artículo*). For example, a plaintiff may challenge “Article 23 of law X.” Nonetheless, the Supreme Court is not bound to reach integer values per normative provision. For example, Article 23 may have two paragraphs, and the first one may be declared constitutional while the second one may be invalidated. Given the classification values explained above, the court may reach either absolute categories within a single provision or up to four different partial results involving a combination of the above categories. To quantify the above situation effectively, integer results were assigned the numerical value of 1. In contrast, partial results were assigned a fractional value corresponding to the number of competing results from the same normative provision. If a provision was declared partially constitutional and partially unconstitutional, the dataset registers each result with a .5 value. If a normative provision was partially dismissed on procedural grounds, partially invalidated, and partially upheld, 0.33 is the value assigned. The last scenario—four simultaneous partial results—did not occur in the coding, but it is not uncommon for this jurisdiction, as proven by other datasets on the Mexican Supreme Court (see the supplementary material to [18]). Given the coding of .33 and .5 values, the sum of provisions referring to articles invalidated, upheld, dismissed on procedural grounds, and dismissed due to the supermajority rule may not be an integer.

9-11. Categories 9-11 describe the parties to the case. The plaintiff can be categorized as a Municipality (M), State (S), or Federal Government (F), with the same category repeating regarding the level of government sued. Category 11 indicates in a binary manner whether or not the former Federal District was a party to a case (the Federal District attained the name of “Mexico City” in 2016 through a constitutional amendment, but the dataset maintained the coding as Federal District to ensure consistency). The Federal District, currently Mexico City, is territorially subdivided into boroughs (*delegaciones*) roughly equivalent to municipalities. (0) describes the absence of the Federal District as a party, while (1) denotes it as a party. If a case is coded as (1), it represents that both parties belong to the Federal District/Mexico City (for example, Federal District vs. *Delegación*/Municipality of the Federal District). The only exception is when the conflict involves the Federal Government, in which case only one party, either the plaintiff or sued party, will belong to the Federal District.

12. Category 12 describes the remedy that a party may pursue. According to Article 105 of the Mexican Constitution, an eight-vote supermajority out of the eleven-member court is required to invalidate legislation with *erga omnes* effects, specifically pertaining to cases in which a higher level brings forth a claim against a lower level of government—i.e., Federal Government v. State, Federal Government v. Municipality or State v. Municipality. The Constitution did not specify the majority required in the remaining cases in which effects are only *inter partes*. Since judgment CC 66/2002, the Court interpreted that regardless of the remedy possible, an eight-vote supermajority was required to pursue *erga omnes* or *inter partes* effects. Said supermajority is only applicable to normative provisions. Understanding the relationship between the parties, majorities, and effects is crucial in handling and interpreting the data and determining the remedies the court may issue. The said relationship is portrayed in Table 1.

**Table 1:** Typology of effects and remedies in constitutional controversies.

Conflict as per parties	Constitutional Basis	Type of relationship	Majority required	Remedy Possible
F vs S	105 I, a)	D	8 votes	*erga omnes*
F vs M	105 I, b)	D	8 votes	*erga omnes*
S vs F	105 I, a)	UP	8 votes	*inter partes*
S vs S	105 I, d)	H	8 votes	*inter partes*
S vs M	105 I, i) y j)	D	8 votes	*erga omnes*
M vs M	105 I, g)	H	8 votes	*inter partes*
M vs S	105 I, i) y j)	UP	8 votes	*inter partes*
M vs F	105 I, b)	UP	8 votes	*inter partes*

F: Federal Government; S: State/Federal Entity; M:Municipality; D: Downward; UP: Upward; H: Horizontal

13. Category 13 describes the type of normative provision challenged. (S) comprehends statutory (laws) or sub-statutory provisions, also known as “*reglamentos*.” (CA) denotes constitutional amendments, and (IT) describes international treaties.

Table 2 provides an example of the visual configuration of data:

**Table 2:** Visual example of the Data Display of randomly selected entries (14/688 entries).

Judgment ID	Year Filed	Year Resolved	Supermajority Dismiss	Upheld	Dismissed FG	Unconst.	Total	Plaintiff	Level sued	FD	Effects	Type
352–2001	2001	2002	0	0	5	0	5	M	F	0	1	CA
365–2001	2001	2002	0	0	5	0	5	M	F	0	1	CA
8–2002	2002	2005	0	19	0	0	19	M	S	0	1	S
12–2002	2002	2005	0	49	2	1	52	M	S	0	1	S
13–2002	2002	2003	0	0	0	1	1	M	S	0	1	S
14–2002	2002	2003	0	0	0	1	1	M	S	0	1	S
18–2002	2002	2002	0	1	0	0	1	M	S	0	1	S
19–2002	2002	2002	0	1	0	0	1	M	S	0	1	S
20–2002	2002	2002	0	0	1	0	1	M	S	1	1	S
25–2002	2002	2002	0	0	7	1	8	M	S	0	1	S
27–2002	2002	2003	0	0	0	1	1	M	S	1	1	S
28–2002	2002	2003	0	0,5	0	1,5	2	M	S	1	1	S
29–2002	2002	2003	0	0	0	1	1	M	S	1	1	S
33–2002	2002	2004	0	0	1	1	2	S	F	1	1	IT

Data was acquired on June 22, 2022 and manually classified during the period 22.06.2022-20.07.2022. During August 2024, data was re-cleansed and subjected to additional cross revisions

## Limitations

The dataset is constrained by the spatial scope of data collection, not covering beyond 21.06.2022, after which a period of polarization between the *Movimiento de Regeneración Nacional* (MORENA) parliamentary majority and the Federal Judiciary [22] would make it valuable to test some hypotheses. It must be noted that in September 2024, the "*Reforma Judicial*" (Judicial Reform) was approved, effectively "packing" the Federal Judiciary and the Supreme Court under the pretext of introducing judicial elections. The judicial overhaul is set to significantly reshape the structure of the Supreme Court and the composition of the judiciary. Furthermore, the Mexican Supreme Court resolves cases in public deliberation sessions [23] but issues the written judgment on a later day without a specific mandatory period for it to be formally issued—a process known as “engrosar.” Therefore, it is possible that some cases resolved within the dataset period are not collected and classified since, during the retrieving phase, they might not have been published and available in the Court's database despite having been voted on and resolved.

Finally, individual provision coding may have two additional limitations. Although the dataset covers nuanced results per provision, it does not provide a qualitative analysis of them —for example, their importance to the case or their centrality to the plaintiff. Determining the case's “winner” when several provisions were challenged and the court assigned them different outcomes might be problematic without qualitative analysis. A last limitation is that challenges on the grounds of lack of competence of the legislative body or the unconstitutionality of the legislative procedure, even if affecting all normative provisions issued during said procedure, were coded as a single challenge.

## Ethics Statement

The present study has not involved studies with animals or humans. The author confirms adhering to the guidelines for authors as provided by Data in Brief pertaining to ethical considerations

## CRediT Author Statement

This is a single-authored article: Conceptualization, Investigation, Data curation, Writing–review, and editing were performed by Mauro Arturo Rivera León

## Data Availability

ZenodoFederalist conflicts on the Constitutionality of Laws 1995-June 21 2022 Dataset (Original data)
